# Fixation mechanisms of nanoparticles on substrates by electron beam irradiation

**DOI:** 10.3762/bjnano.8.153

**Published:** 2017-07-26

**Authors:** Daichi Morioka, Tomohiro Nose, Taiki Chikuta, Kazutaka Mitsuishi, Masayuki Shimojo

**Affiliations:** 1 Department of Materials Science, Shibaura Institute of Technology, 3-7-5 Toyosu, Koto, Tokyo, 135-8548, Japan; 2 Research Center for Advanced Measurement and Characterization, National Institute for Materials Science, 1-2-1 Sengen, Tsukuba, 305-0047, Japan

**Keywords:** accelerating voltage, electron beam, gold, Monte Carlo simulation, nanoparticle array

## Abstract

For applications such as the fabrication of plasmonic waveguides we developed a patterning technique to fabricate an array of nanoparticles on a substrate using focused electron beams (Noriki, T.; Abe, S.;.Kajikawa, K.; Shimojo, M. *Beilstein J. Nanotechnol.* **2015,** *6,* 1010–1015). This technique consists of three steps: Firstly, nanoparticles are placed over the entire surface of a substrate. Secondly, the nanoparticles are fixed on the substrate by focused electron beam irradiation. The electron beam decomposes the organic molecules located around the particle into amorphous carbon. The amorphous carbon immobilizes the particle on the substrate. Finally, the unfixed nanoparticles are removed. However, in this original technique, the area in which the nanoparticles were fixed was wider than the electron-probe size of a few nanometers. To understand this widening mechanisms, the effects of accelerating voltage, particle size and substrate material are investigated by means of both experiments and simulation. It is demonstrated that the fixing area is greatly affected by the electrons back-scattered by the substrate. The back-scattering leads to an increase in line width and thus reduces the resolution of this patterning technique.

## Introduction

Techniques to fabricate assemblies or arrays of nanostructures on a desired area have been attracting attention because these arrays and patterns offer unique electrical and optical properties. One of the applications of such nanostructure arrays is plasmonic waveguides, in which the energy of light propagates because of the localized surface plasmon resonance (LSPR) effect [1–2]. In particular, arrays of gold or silver nanostructures can be used for such waveguides, as nanostructures made of these materials interact with visible light. Such LSPR structures would make the development of smaller optical circuits and devices possible.

Plasmon propagation through nanowires or rows of nanoparticles was studied by several researchers [3–5]. However, in most of the experiments nanowires or nanoparticles were deposited on substrates without attempting to control their positions. Therefore, a practical technique is necessary to produce nanoparticle patterns. Rows of nanoparticles could be produced by focused electron beam induced deposition (FEBID) [6], photo-lithography (PL), or micro-contact printing (μCP) [7]. However, the purity of the deposits from FEBID is generally low, and PL and μCP require complicated processes including the fabrication of masks or masters, exposure or stamping, and several steps of chemical treatments.

Noriki et al. [8] combined electron beam irradiation with a chemical reaction to pattern gold nanoparticles onto substrates. This technique consists of three steps: Firstly, gold nanoparticles are placed over the entire surface of a substrate. Secondly, the gold nanoparticles are fixed on the substrate by electron beam irradiation. Finally, the unfixed nanoparticles are removed. In the second step, the organic molecules (e.g. citrate) surrounding the nanoparticles are decomposed to amorphous carbon, and this amorphous carbon existing in the gap between the particle and the substrate fixes the particles. However, in this original technique, the area of fixed gold nanoparticles was wider than the electron-probe size of a few nanometers [8]. To understand the mechanisms of this widening, the effects of accelerating voltage, particle size and substrate are investigated by means of both experiments and Monte Carlo (MC) simulation in this study.

## Result and Discussion

**Figure 1:**
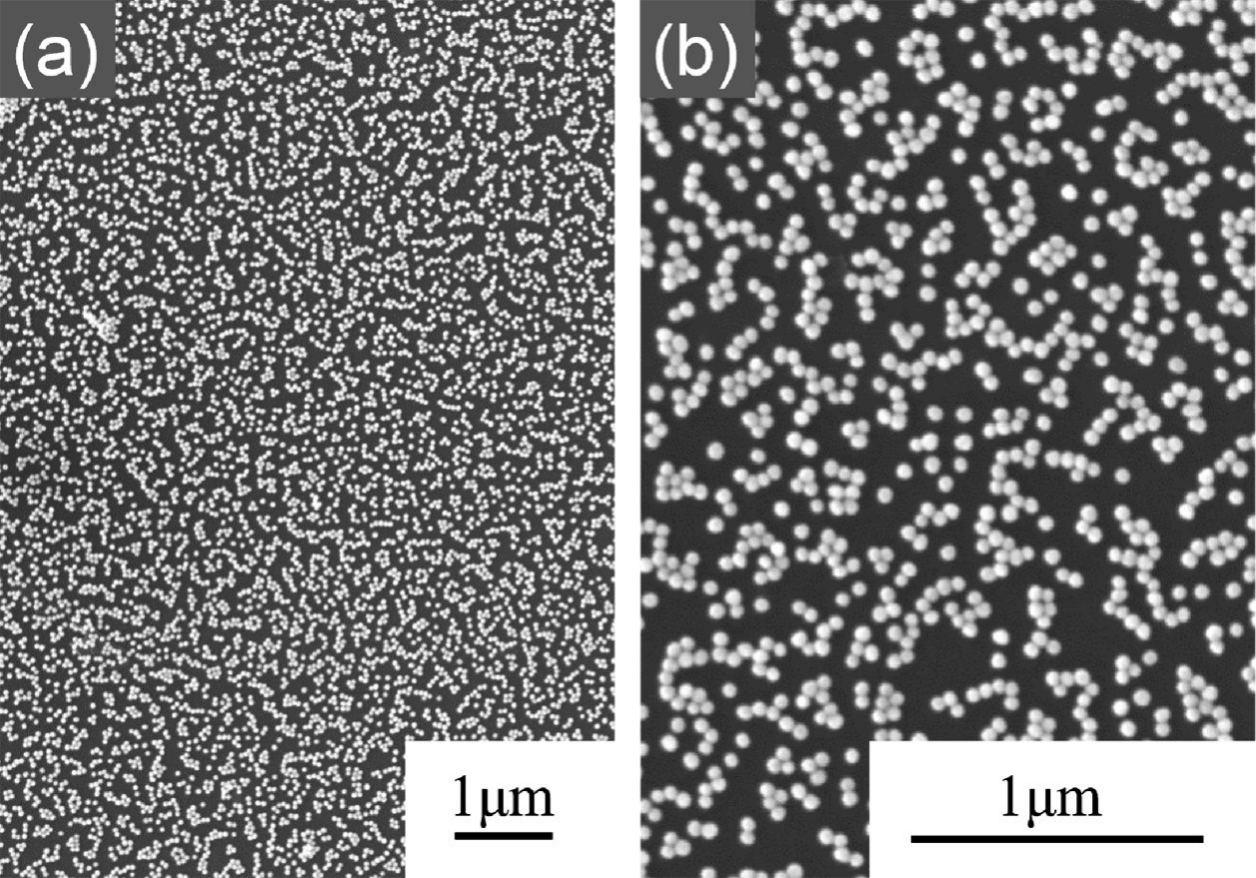
SEM images of gold nanoparticles (diameter = 50 nm) placed over the Si substrate in the first step, taken at (a) low and (b) high magnification.

In the first step of the experimental procedure, gold nanoparticles were placed over the substrate by immersing substrates in a colloidal gold solution for 24 h. Figure 1 shows scanning electron microscopy (SEM) images of the gold nanoparticles, the diameter of which was 50 nm for these images, on a silicon substrate. The particles arranged two-dimensionally without three-dimensional aggregation.

**Figure 2:**
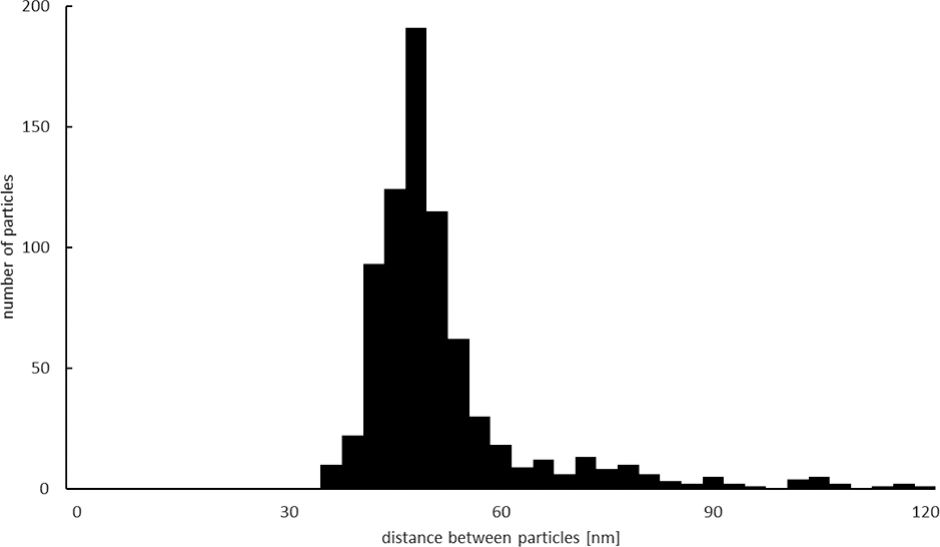
Distribution of the distance between particles.

As the distance between particles is important for plasmonic coupling, the distribution of the center-to-center distance of the nearest particles measured from SEM images is shown in Figure 2. This distribution may be controlled by changing the immersing time in the colloidal solution.

### Effect of particle size

**Figure 3:**
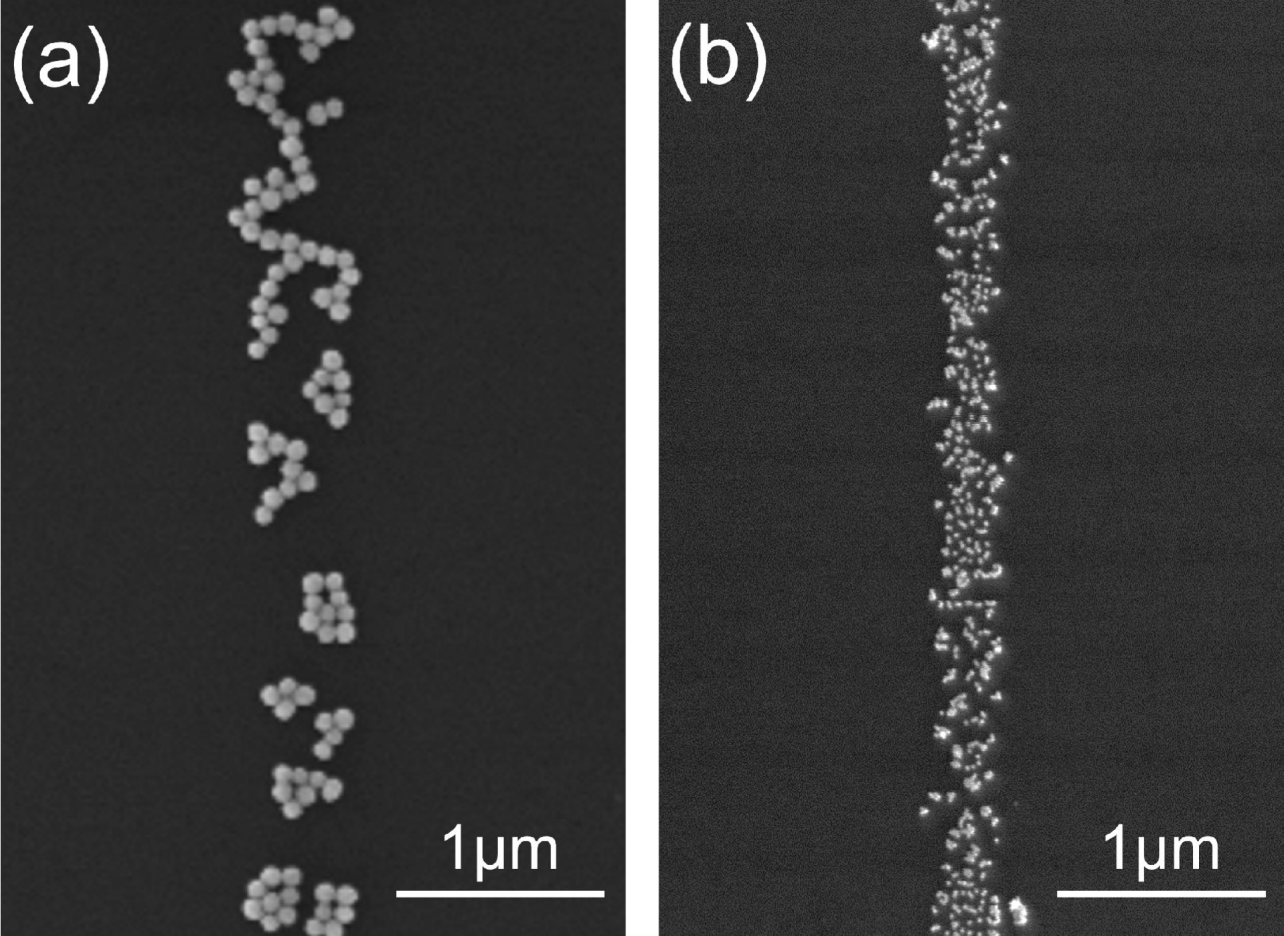
SEM images of gold nanoparticles, the diameters of which are (a) 100 nm and (b) 20 nm, fixed on the Si substrate at an accelerating voltage of 2 kV, a probe current of 3 × 10^−11^ A and a scan speed of 0.7 μm/s.

Figure 3 shows scanning electron microscopy (SEM) images of gold nanoparticles, the diameters of which are 100 nm and 20 nm, fixed on the Si substrate by a line scan of the electron beam at an accelerating voltage of 2 kV. The particles were fixed on lines with finite widths. The widths of the particle lines are 0.5 μm and 0.3 μm for particle sizes of 100 nm and 20 nm, respectively. This suggests that an increase in particle size increases the line width. This is considered to be due to the scattering of electrons in the particles.

**Figure 4:**
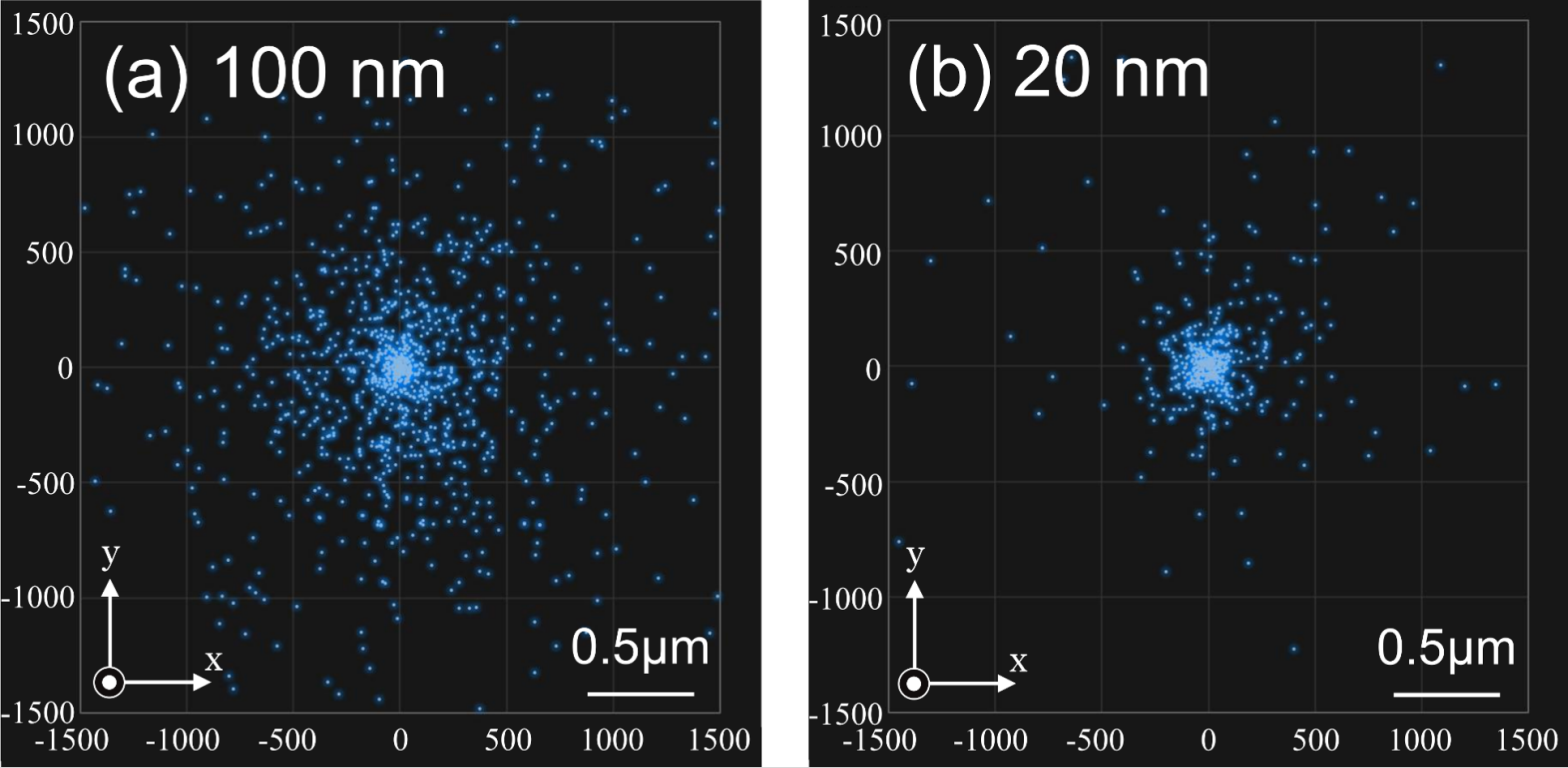
Distribution of points at which electrons arrive at the substrate surface due to scattering in the Au particle calculated by Monte Carlo modeling. The particle sizes are (a) 100 nm and (b) 20 nm. The initial electron energy is 2 keV. The number of irradiated electrons per unit length is 500 nm^−1^.

Figure 4 shows MC simulation results indicating the positions of electrons arriving at the substrate surface after scattering in a particle, simulated with the same particle sizes and accelerating voltage, i.e., initial electron energy, as those of Figure 3. This also indicates that the larger the particle size, the longer distance or range the electrons scatter. According to statistical analysis, about 70% of electrons arriving at the surface are concentrated in a diameter of 1.0 μm from the origin after being scattered in a 100 nm particle. After being scattered in a 20 nm particle, the electrons are concentrated in a diameter of 0.30 μm. As the initial beam position is scanned in a line over the particles, each distribution should be elongated about 100 nm or 20 nm along the scanning direction. However, this is negligible at this scale.

### Effect of electron-beam condition

**Figure 5:**
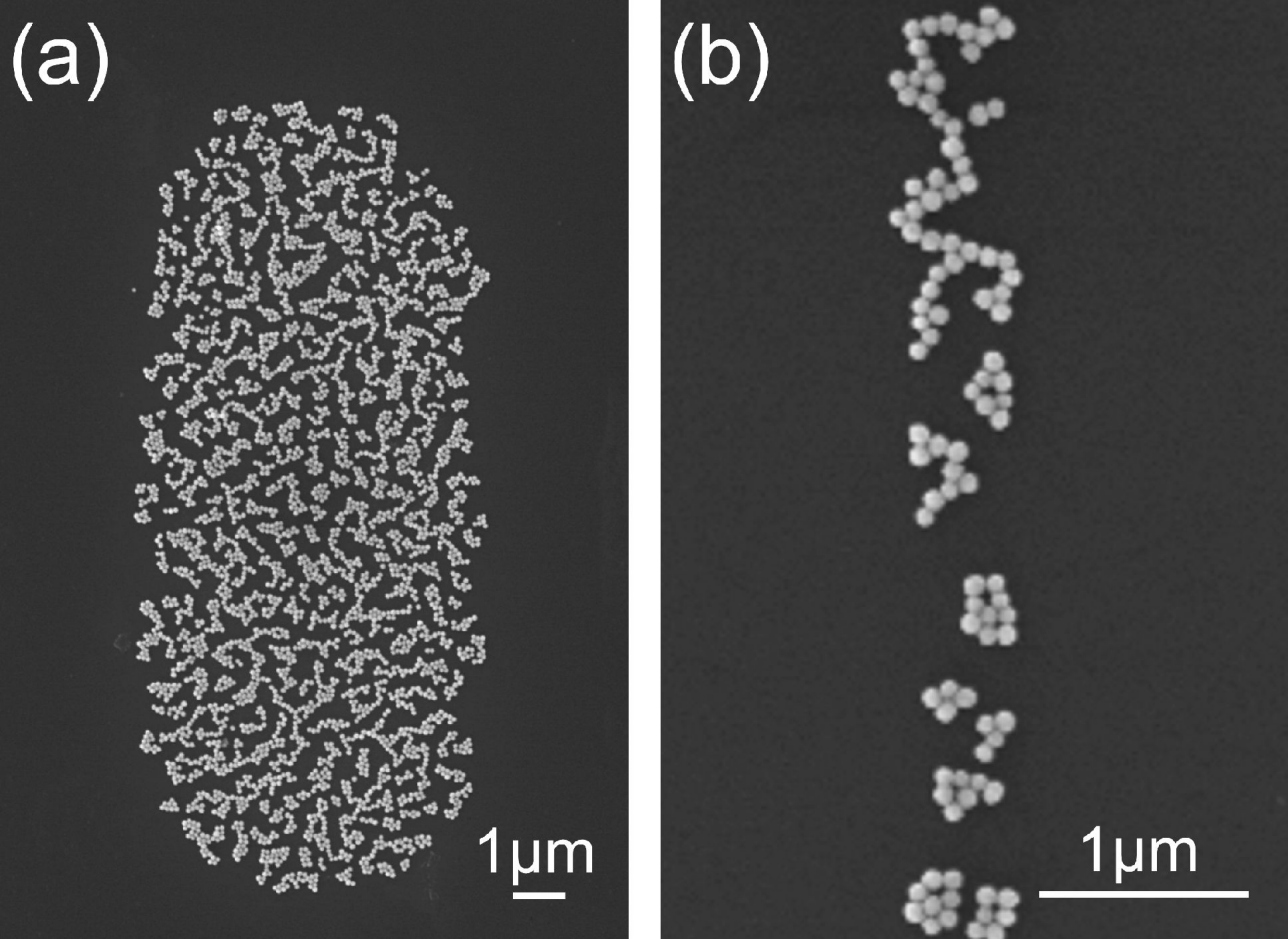
SEM images of gold nanoparticles, the diameter of which is 100 nm, fixed on the Si substrate at accelerating voltages of (a) 20 kV and (b) 2 kV, a probe current of 3 × 10^−11^ A and a scan speed of 0.7 μm/s. Note that image (b) is the same as Figure 3a.

Figure 5 shows SEM images reflecting the effects of the accelerating voltage on the line width. The width was about 6.5 μm at an accelerating voltage of 20 kV, whereas it was 0.5 μm at 2 kV. The widening of the line width due to the high accelerating voltage is much larger than that due to large particle size described above.

**Figure 6:**
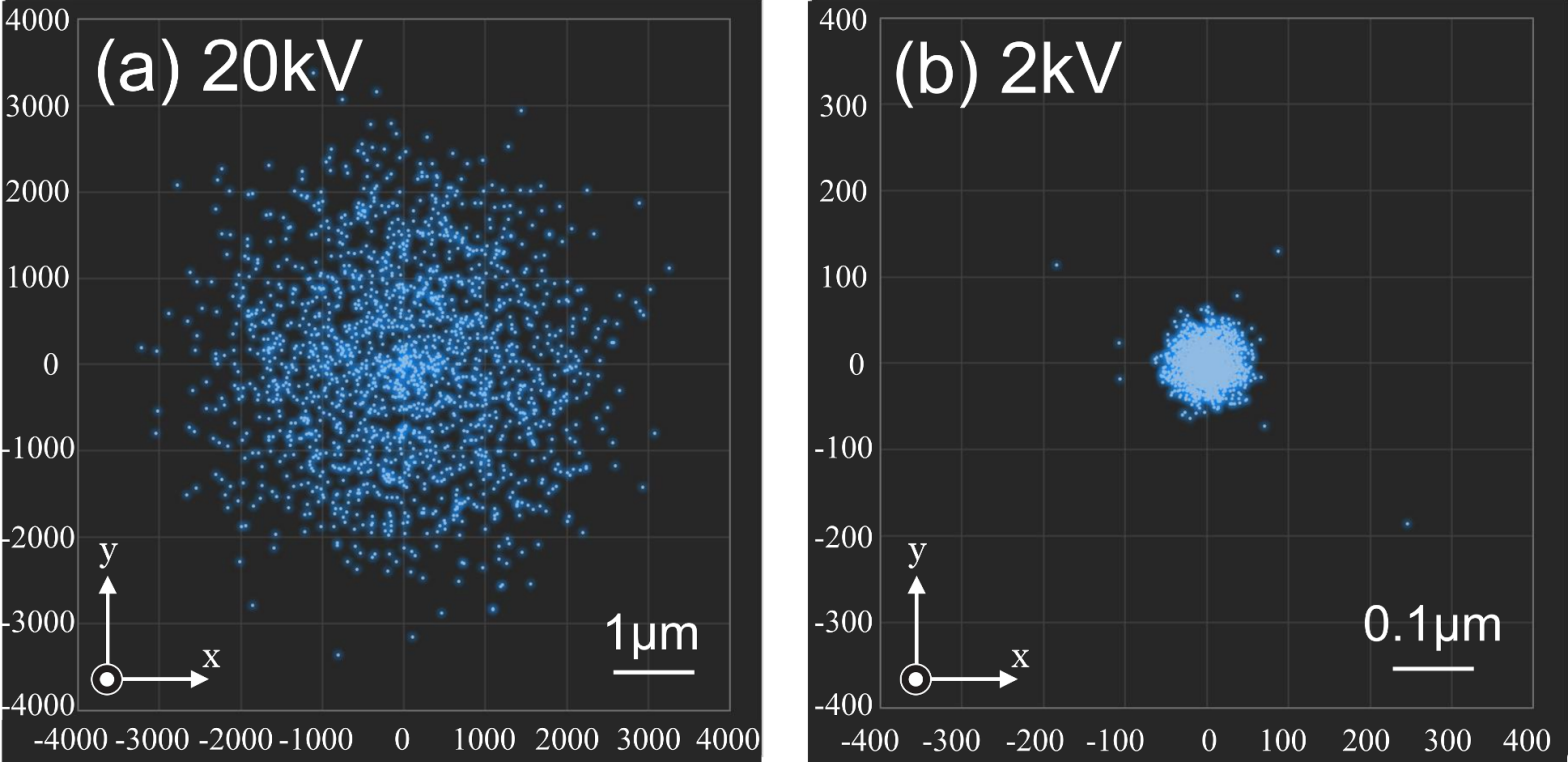
Distribution of points, at which electrons arrive at the substrate surface due to back-scattering in the Si substrate, calculated by Monte Carlo modeling. The initial electron energies are (a) 20 keV and (b) 2 keV. The number of simulated electrons is 10000.

Figure 6 shows MC simulation results of the distribution of electrons arriving at the substrate surface after being scattered in the substrate at accelerating voltages of 20 kV and 2 kV. Most electrons arrive at the surface in a diameter of 6.0 μm at 20 kV, and 0.1 μm at 2 kV, respectively. This is in good agreement with the experimental results shown in Figure 5. This suggests that the widening is caused mostly by the electron scattering in the substrate. The backscattering ranges were theoretically analyzed for several materials at various electron energies by Kanaya [9], and the results agree well with our MC results.

**Figure 7:**
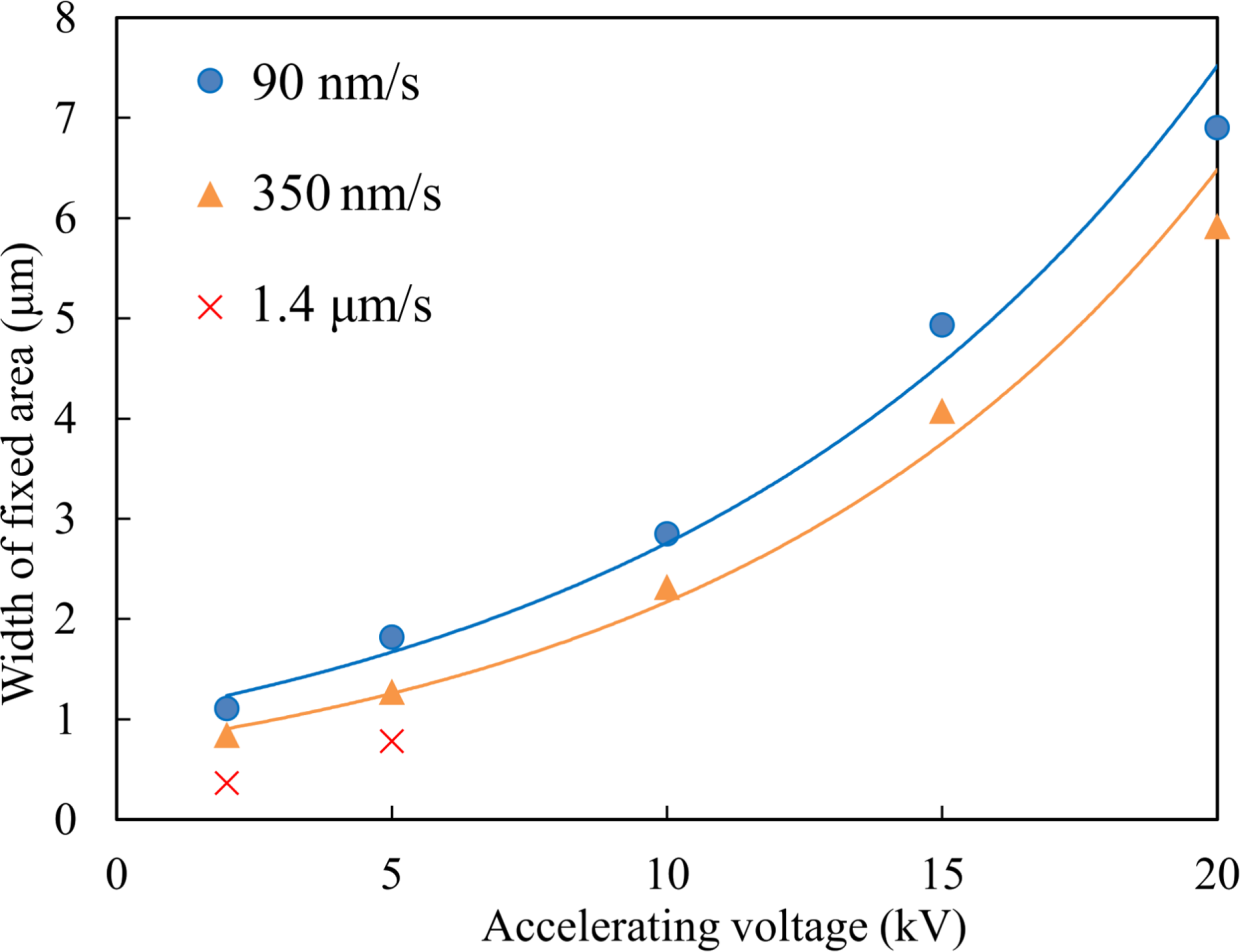
Width of fixing area as a function of accelerating voltage and scan speed, at a probe current of 3 × 10^−10^ A, and for a particle size of 100 nm, measured from experiments.

The effects of electron-beam conditions including accelerating voltage and beam scan speed, obtained by experiments, are summarized in Figure 7. The width increases greatly with increasing accelerating voltage. An increase in width with decreasing scan speed is reasonable because the number of irradiating electrons per unit area increases.

**Figure 8:**
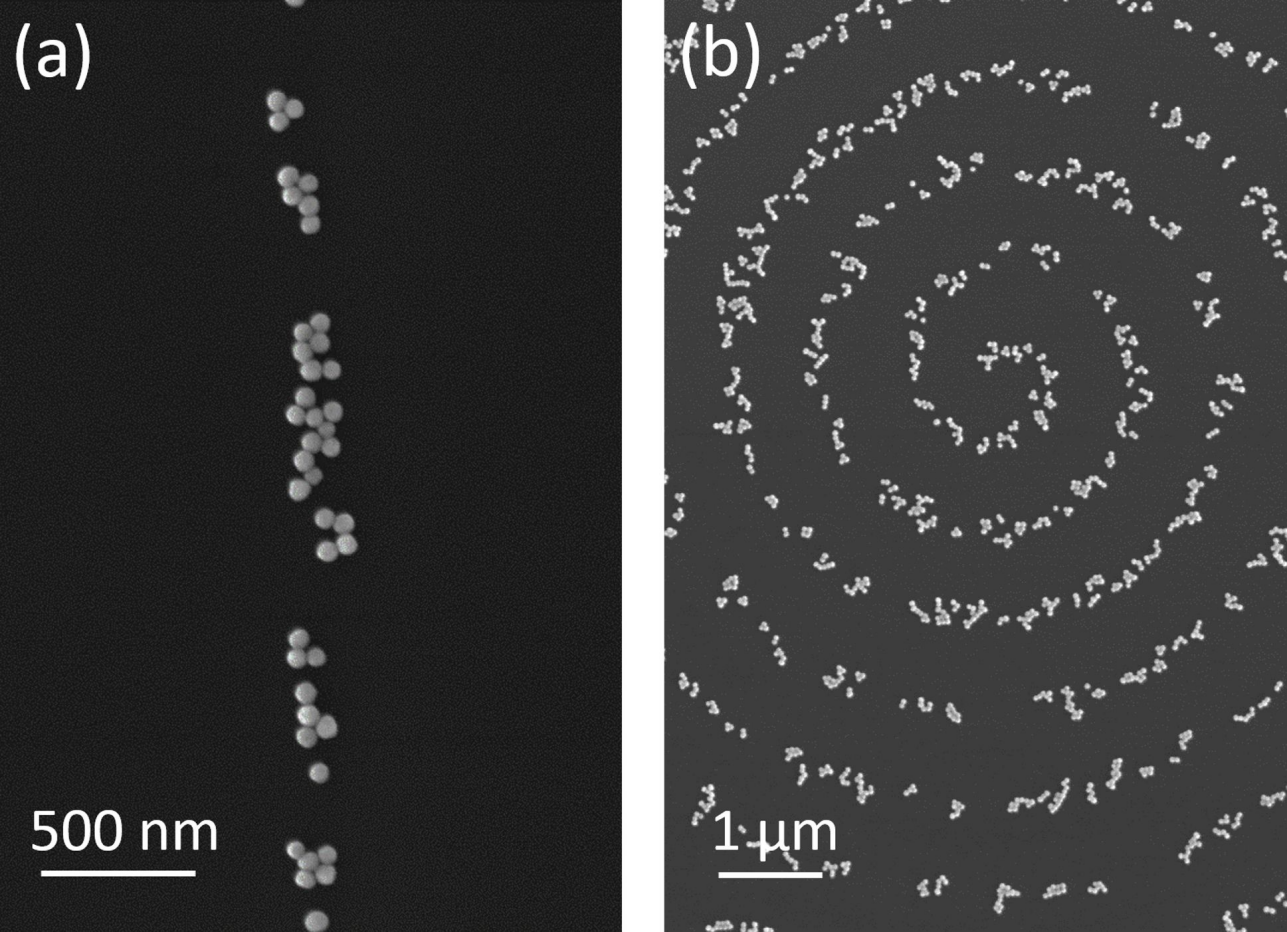
SEM images of gold nanoparticles, the diameter of which is 50 nm, fixed by (a) a line scan and (b) a spiral scan of electron beams at accelerating voltages of 1.5 kV for (a) and 2 kV for (b), at a probe current of 3 × 10^−10^ A, and a scan speed of 1.4 μm/s.

To achieve thin lines, decreasing the accelerating voltage and increasing the scan speed are thus effective. Figure 8a shows a line of particles produced at an accelerating voltage of 1.5 kV and a scan speed of 1.4 μm/s, resulting in a line width of about 0.13 μm. Figure 8b shows a spiral pattern of a particle line demonstrating the flexibility of this technique.

### Effect of substrates

**Figure 9:**
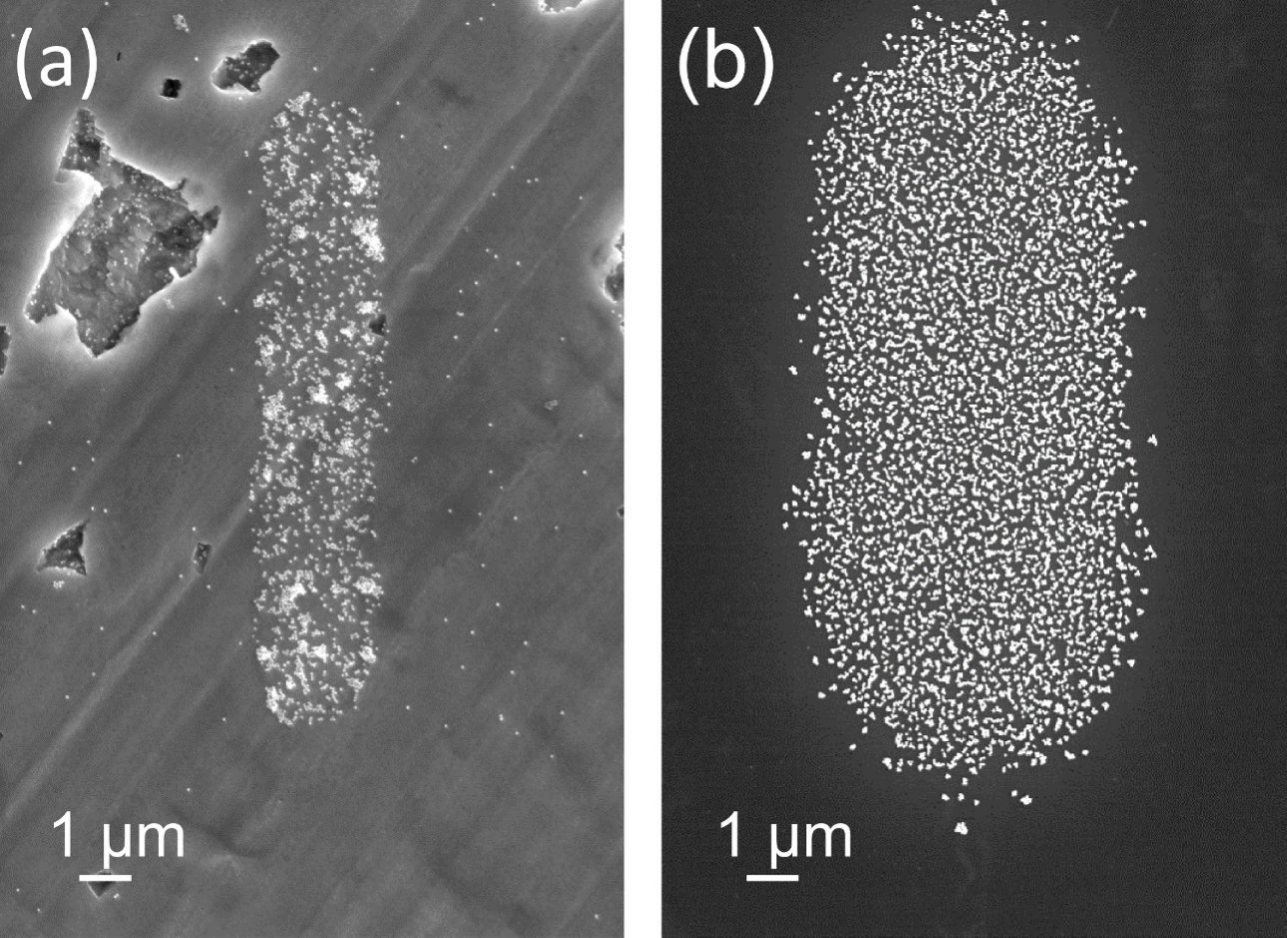
SEM images of gold nanoparticles fixed on (a) Cu and on (b) Si substrates, at an accelerating voltage of 20 kV, a probe current of 3 × 10^-11^ A, and a scan speed of 0.35 μm/s.

Figure 6 suggested that the widening was caused by the scattering of electrons in the substrate. Thus, the effect of the substrate material is investigated. Figure 9 shows lines of particles produced on Cu and Si substrates. The widths are 2.3 μm and 6.2 μm for Cu and Si substrates, respectively. This indicates that the scattering distance becomes shorter for higher substrate densities or higher atomic numbers, as indicated frequently in textbooks of SEM [10].

**Figure 10:**
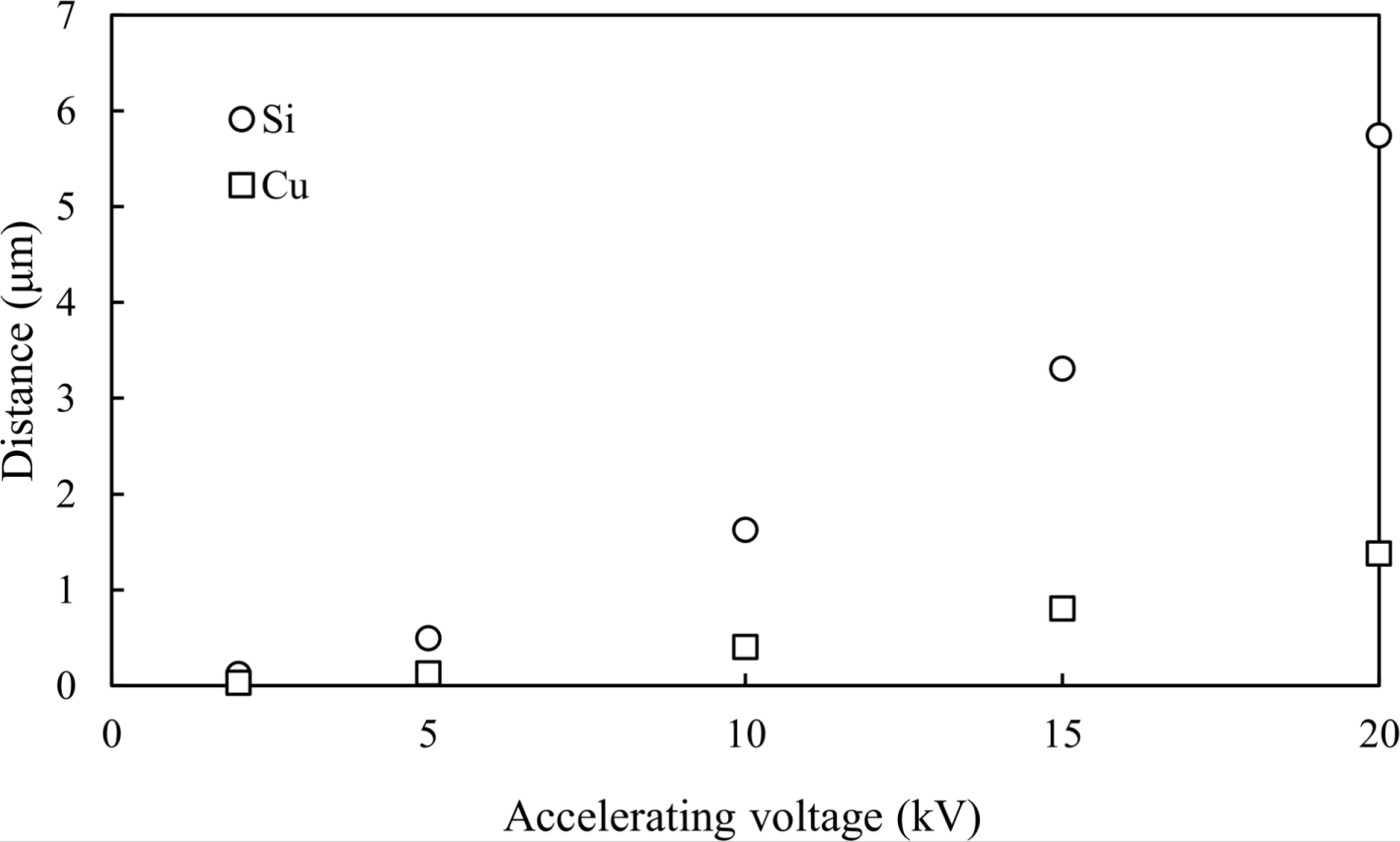
Distance of scattered electron arriving at the surface of the substrate calculated by Monte Carlo modeling.

Figure 10 shows the distance of scatter, which is defined as 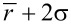 (where 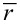 is the average of the distance *r* between the irradiation point and the point which the scattered electron arrives at the substrate surface, and σ is the standard deviation of *r*), as a function of the accelerating voltage, i.e., the initial electron energy, for two substrates calculated by MC modeling. This result also supports the conclusion that the widening is caused by the scattering of electrons in the substrate.

### Silica nanoparticles

**Figure 11:**
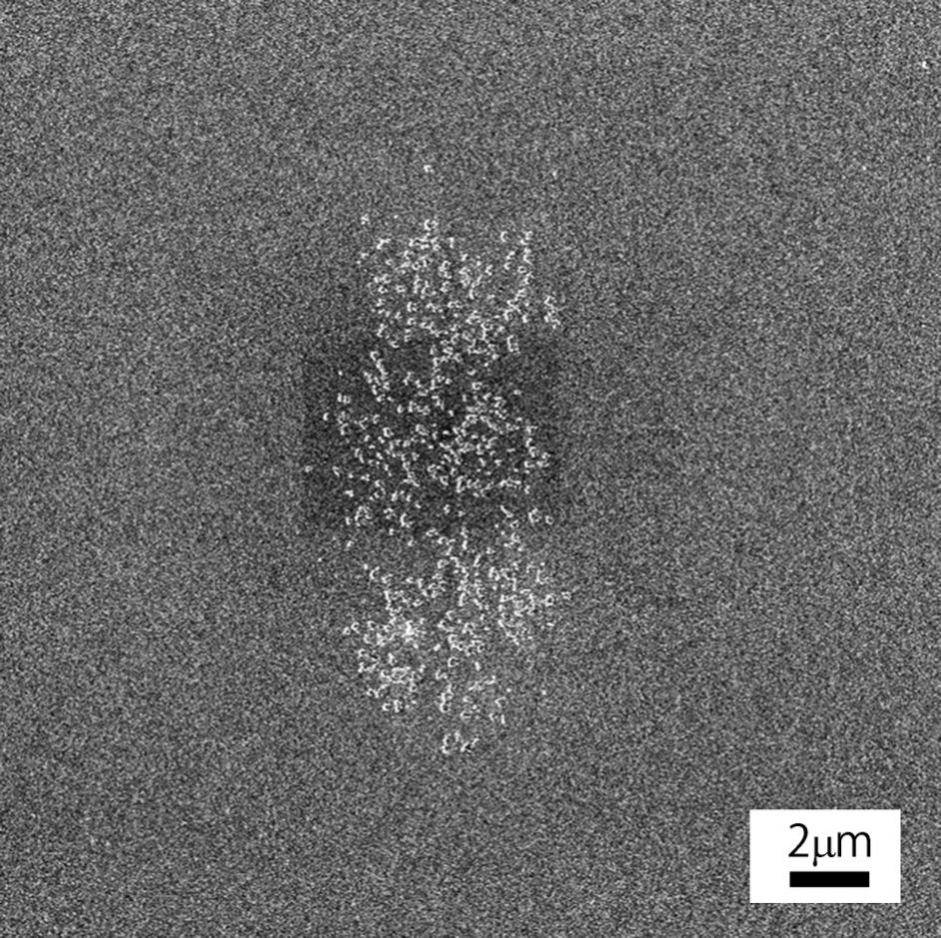
SEM image of silica nanoparticles fixed on a Au-coated Si substrate.

Electron beam induced fixation can be applied not only to Au but also to other materials. To indicate the versatility of this technique and the possibility of other applications, the fixation of silica nanoparticles was also demonstrated. Figure 11 shows silica nanoparticles fixed on a Au-coated Si substrate. As the surface of colloidal silica particles was modified with –COOH groups, a dissociation of the organic shells occurs and the particles are fixed on the substrate.

## Conclusion

The mechanism of fixing nanoparticles and the width of the fixing areas in the technique, proposed by T. Noriki et al. [8], to fabricate gold nanoparticle arrays on substrates are studied by means of both experiments and simulation. The widths are measured and calculated with changing the accelerating voltage, particle size and substrate material.

The width depends greatly on the accelerating voltage of incident electrons, substrate material, and slightly on the diameter of particles. These results suggest that the width is affected largely by back-scattering from the substrate and partly by scattering in the particle.

## Experimental

### Experimental procedure for the measurement of fixing width

Citrate stabilized colloidal gold nanoparticles, the diameters of which were 20–100 nm, were purchased from Tanaka Holdings Co., Ltd., Japan. A polished Si wafer (p-type), with dimensions of about 4 × 1 mm^2^, was used as substrates.

**Figure 12:**
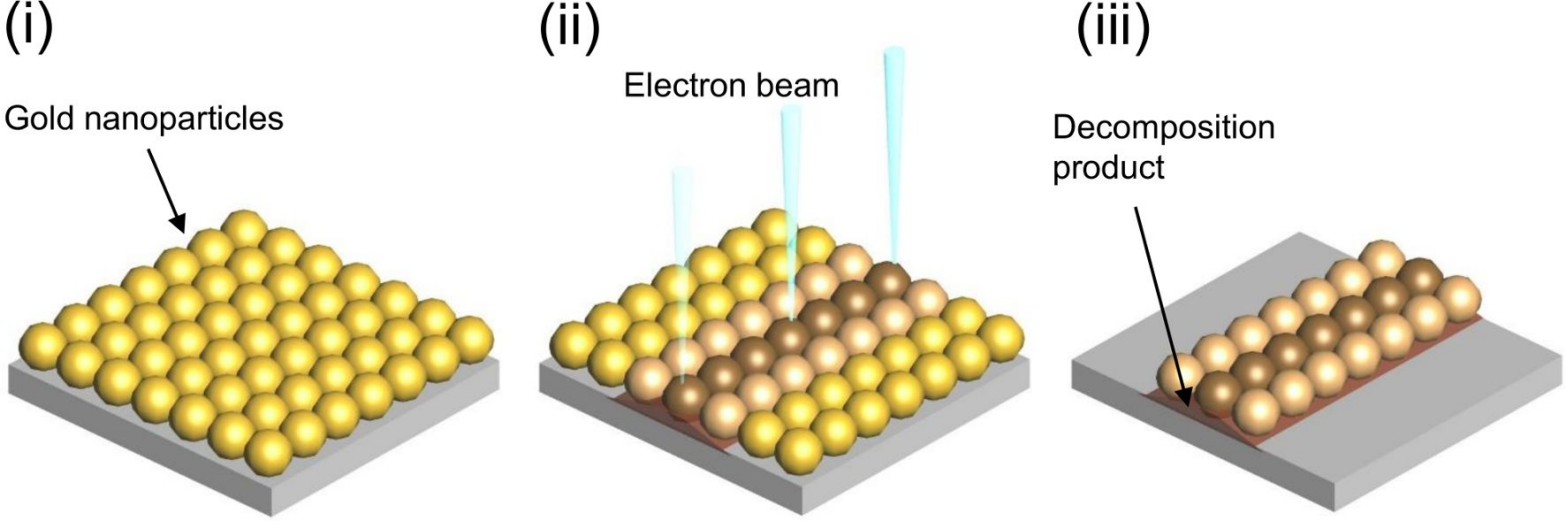
Schematic illustration of the experimental procedure consisting of (i) the arrangement of gold nanoparticles, (ii) the scan of electron beam, and (iii) the removal of unfixed particles.

A schematic illustration of this experimental technique is shown in Figure 12. A Si substrate was immersed in the colloidal Au solution for 24 h at room temperature to place nanoparticles uniformly over the surface of the substrate. Then a focused electron beam was scanned in a line over the substrate with nanoparticles using a field-emission scanning electron microscope (SEM, JEOL JSM7800-UHV), to immobilize particles on the substrate. The fixing mechanisms are described elsewhere [8]. The substrate was rinsed using ultra-sonication in an ethanol solution of dodecanethiol for 10 min to remove unfixed particles. Dodecanethiol and ethanol were purchased from Wako Co., Ltd., Japan. The substrate was observed with SEM and the width of the area in which the nanoparticles remained was measured.

To show the versatility of this technique silica nanoparticles were also fixed. An Au thin film, the thickness of which was about 5 nm, was deposited by sputtering on the same Si substrate as used for Au nanoparticles. The surface of the Au-deposited substrate was covered with a monolayer of amino-undecanethiol. Then, the substrate was immersed in a colloidal silica (surface-modified with –COOH) solution for 24 h to arrange nanoparticles as the first step. The diameter of the silica particles was 100 nm. An electron beam (20 kV, 3 × 10^−10^ A) was scanned in a line of 10 μm in length at a scanning speed of 0.1 μm/s. Finally, the substrate was washed in ethanol for 5 min.

### Monte Carlo simulation of scattering electrons for the calculation of fixing area

The Monte Carlo method is a technique to reproduce a physical situation using random numbers [11]. In short, the basics of the simulation, the details of which are written elsewhere [11–12], are as follows: The relation between the mean free path λ and the scattering cross section σ_E_ is

[1]
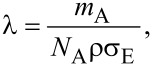


where *N*_A_ is Avogadro’s number, ρ is the density of the material, and *m*_A_ is the atomic weight of the material. The step distance *s*, which is the distance between two scattering events, is considered to be a random value keeping the average step distance equal to the mean free path,

[2]
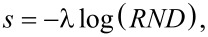


where *RND* indicates a random number between 0 and 1 generated by the computer.

The scattering angle θ is derived from the Rutherford cross-section as

[3]
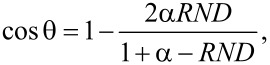


where α = 0.034·[*Z*^2/3^/*E*(keV)], *E* is the energy of the electron, and *Z* is the atomic number of the material.

The azimuthal angle of scatter φ is considered to be random:

[4]
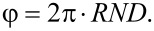


The average energy loss at each scattering event, which is called stopping power, is

[5]
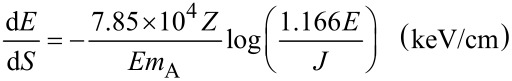


where *J* is the mean ionization potential given by *J* = 9.76*Z* + 58.5*Z*^−0.19^, and *S* is the product of ρ and *s*.

**Figure 13:**
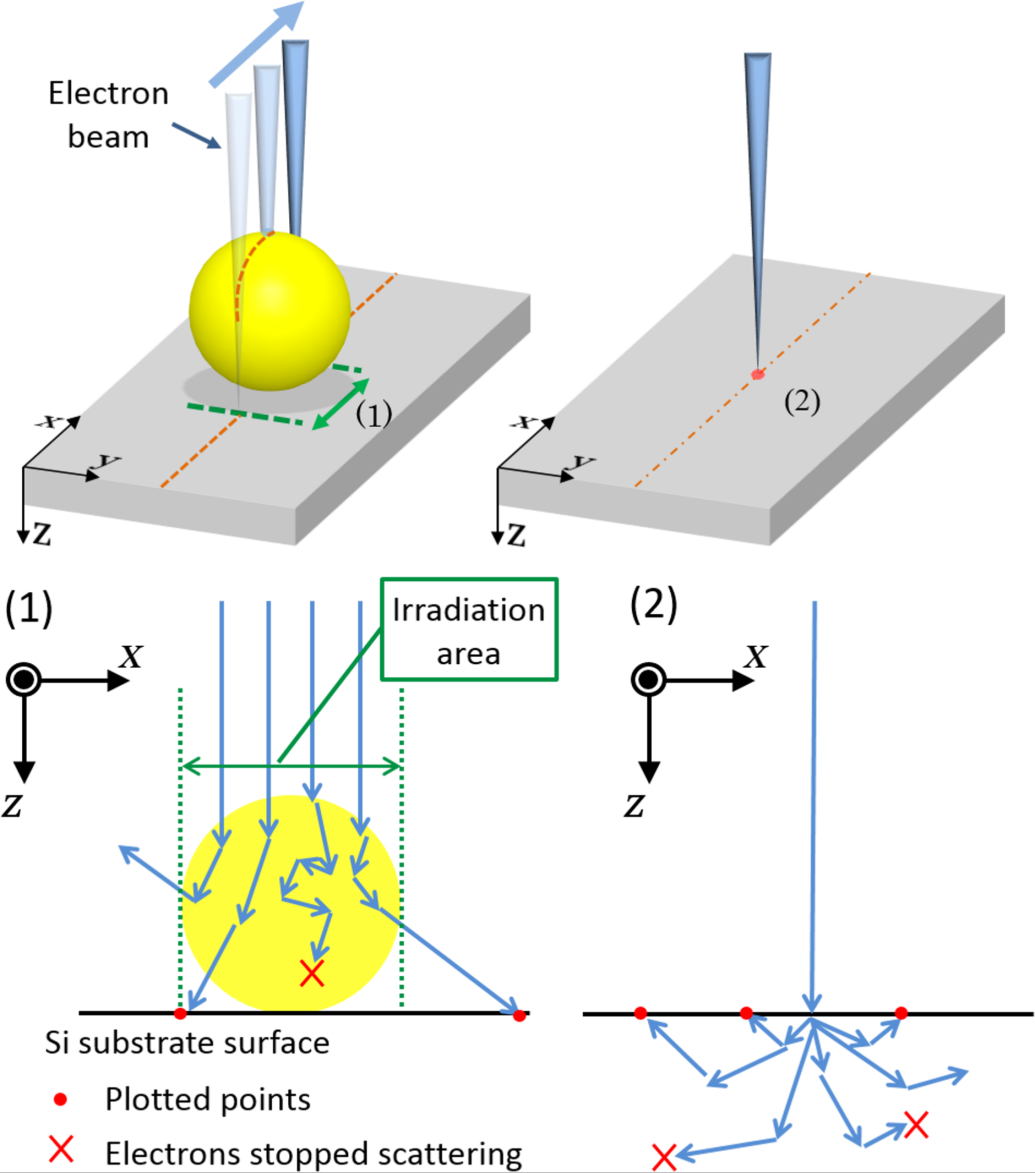
Schematic illustration of the calculation of electron trajectories using Monte Carlo modeling: (1) electrons incident to a gold nanoparticle; (2) electrons incident to the Si substrate.

We have developed a software written in C# language with reference to the Monte Carlo simulation modeling for electron trajectories proposed by D. C. Joy [12] and K. Mitsuishi [11], the summary of which is written above. The electron trajectories were calculated for electrons entering the particles or entering the substrate, with changing the electron energy, particle size and substrate material corresponding to the experimental conditions. The schematic illustration for this simulation is shown in Figure 13. For the case of electrons entering the particle, the calculations were performed at several incident positions on a line from one end to another end across the center, as shown in Figure 13(1), with a density of 500 electrons/nm. For the case of electrons entering the substrate, the calculations were repeated for 10000 electrons. The electron energy, i.e., the accelerating voltage of the incident electrons, is decreased at each scattering process in the material (either particle or substrate), according to the stopping power described above. The calculation was stopped when the electron energy became 35 eV or less. The positions at which the scattered electrons arrive at the surface of the substrate are the positions at which the fixation reaction is considered to occur at a certain probability. The size of the electron beam was assumed to be zero in the MC simulation. Although the actual beam diameter used for the experiments was a few nanometers, the assumption of a zero-diameter beam is reasonable, because the range of scatter was of the order of micrometers, which is much larger than the actual beam size.
